# Supporting Harm Reduction through Peer Support (SHARPS): testing the feasibility and acceptability of a peer-delivered, relational intervention for people with problem substance use who are homeless, to improve health outcomes, quality of life and social functioning and reduce harms: study protocol

**DOI:** 10.1186/s40814-019-0447-0

**Published:** 2019-04-29

**Authors:** Tessa Parkes, Catriona Matheson, Hannah Carver, John Budd, Dave Liddell, Jason Wallace, Bernie Pauly, Maria Fotopoulou, Adam Burley, Isobel Anderson, Graeme MacLennan, Rebecca Foster

**Affiliations:** 1 Salvation Army Centre for Addiction Services and Research, Faculty of Social Sciences, University of Stirling, Colin Bell Building, Stirling, FK9 4LA UK; 2 Edinburgh Access Practice, NHS Lothian, 22-24 Spittal Street, Edinburgh, EH3 9DU UK; 3 Scottish Drugs Forum, 91 Mitchell Street, Glasgow, G1 3LN UK; 4 Canadian Institute for Substance Use Research, Technology Enterprise Facility, University of Victoria, Room 273, Victoria, British Columbia V8P 5C2 Canada; 5 Faculty of Social Sciences, University of Stirling, Colin Bell Building, Stirling, FK9 4LA UK; 6 The Access Point, 17/23 Leith Street, Edinburgh, EH1 3AT UK; 7 The Centre for Healthcare Randomised Trials (CHaRT), University of Aberdeen, Health Sciences Building, Foresterhill, Aberdeen, AB25 2ZD UK

**Keywords:** Harm reduction, Substance use, Peer Navigators, Homelessness, Feasibility trial, Intervention

## Abstract

**Background:**

While people who are homeless often experience poor mental and physical health and problem substance use, getting access to appropriate services can be challenging. The development of trusting relationships with non-judgemental staff can facilitate initial and sustained engagement with health and wider support services. Peer-delivered approaches seem to have particular promise, but there is limited evidence regarding peer interventions that are both acceptable to, and effective for, people who are homeless and using drugs and/or alcohol. In the proposed study, we will develop and test the use of a peer-to-peer relational intervention with people experiencing homelessness. Drawing on the concept of psychologically informed environments, it will focus on building trusting and supportive relationships and providing practical elements of support such as access to primary care, treatment and housing options.

**Methods:**

A mixed-method feasibility study with concurrent process evaluation will be conducted to explore the feasibility and acceptability of a peer-delivered, relational intervention for people with problem substance use who are homeless. Peer Navigators will be based in homelessness outreach and residential services in Scotland and England. Peer Navigators will work with a small number of participants for up to 12 months providing both practical and emotional support. The sample size for the intervention is 60. Those receiving the intervention must be currently homeless or at risk of homelessness, over the age of 18 years and self-report alcohol/drug problems. A holistic health check will be conducted in the first few months of the intervention and repeated towards the end. Health checks will be conducted by a researcher in the service where the Peer Navigator is based. Semi-structured qualitative interviews with intervention participants and staff in both intervention and standard care settings, and all Peer Navigators, will be conducted to explore their experiences with the intervention. Non-participant observation will be conducted in intervention and standard care sites to document similarities and differences between care pathways.

**Discussion:**

The SHARPS study will provide evidence regarding whether a peer-delivered harm reduction intervention is feasible and acceptable to people experiencing homelessness and problem substance use in order to develop a definitive trial.

**Trial registration:**

SRCTN registry ISRCTN15900054, protocol version 1.3, March 12, 2018

**Electronic supplementary material:**

The online version of this article (10.1186/s40814-019-0447-0) contains supplementary material, which is available to authorized users.

## Background

Homelessness is a complex issue that often involves deep social exclusion. This is a term which refers to intersections of experiences of homelessness, substance use, institutional care, and ‘street culture’ activities such as begging and street drinking [1]. Estimates suggest that 307,000 people in the UK [2], 550,000 in the USA [3] and 235,000 in Canada [4] experience homelessness at any one point, and these rates have been increasing. Homelessness can be viewed as being caused by ‘individualistic’ or ‘structural’ conditions, with different explanations favoured by different countries [5]. Poverty and other factors, such as traumatic childhood experiences, imprisonment and institutional care, are central to the causes of homelessness [5, 6]. Homelessness can be viewed as both created and exacerbated by systemic changes in housing and social systems, combined with situational factors, that make those with the least power and resources vulnerable to becoming homeless [5].

People experiencing homelessness are vulnerable to ‘tri-morbidity’, with poor mental and physical health and problem substance use [7]. The use of alcohol and drugs is often a factor contributing to someone becoming homeless and can increase as a way of coping with it [8]. People who are homeless often report far worse physical and mental health than the general population [9–11] and are four times more likely to die prematurely and seven times more likely to die as a result of drug use, than the general population [12]. Despite many people who are homeless in the UK being registered with a General Practitioner (GP), a significant number report that they are not receiving help with their health problems [13]: they do not access healthcare services until a crisis emerges, utilising accident and emergency services rather than primary care [7, 14–16]. This can be costly to healthcare funders [7, 17]. Further, when people who are homeless do access mainstream healthcare or substance use services, their needs are not well met. They often experience stigma and negative attitudes from staff, are viewed as second class citizens, and encounter inflexible services that do not meet their needs [14, 15, 18, 19]. Collaborative working between healthcare and housing services is therefore essential [20], and interventions to improve the health of people who are homeless have received increased attention in the last decade [21]. Several systematic reviews have examined the effectiveness of interventions to improve health and substance use outcomes for those who are homeless with findings indicating that having primary care services tailored to those experiencing homelessness [22], case management [9, 21, 23] and provision of housing [21], can be effective in improving mental and physical health and assisting with problem substance use.

In terms of problem substance use, treatment approaches have traditionally been divided into those aimed at helping people stop using alcohol and drugs, with abstinence being the goal, and those taking a harm reduction approach first and foremost [24]. More recently, there has been a move away from understanding these approaches as distinct and separate. That said, there have been questions raised regarding whether or not abstinence-focused interventions are appropriate for people with very complex health and substance use needs [22], such as people who are homeless. While abstinence-based interventions can be effective for some, they rely on people who are homeless having access to services and resources, which can be very difficult. Unstable living conditions can mean that treatment appointments are missed and plans and regimes challenging to maintain [14]. For most people experiencing homelessness who use alcohol and drugs, abstinence is unlikely to be achieved in the short term, so approaches that reduce the harms associated with use are needed [24–26]. It has been recommended that harm reduction approaches be specifically employed to prevent harms related to substance use, with abstinence-based treatment available as an option [25]. Harm reduction aims to support people ‘where they are at’, where substance use is met with a non-judgemental response. Intervention is concerned to prevent harms of use, rather than seeking particular goals [27]. This can facilitate more autonomy because importance is placed on people exercising choice to set their own goals; they are not forced to reduce use or be abstinent [27–29]. Harm reduction services can also act as a ‘gateway’ to other services, including to health and housing services, and specialised substance use treatment [30, 31]. For those who are homeless, there is a need for a wide range of approaches and services to reduce risks including of fatal and non-fatal overdose, including the provision of overdose awareness training and naloxone, heroin-assisted treatment, drug consumption rooms and assertive outreach services [32].

In harm reduction services, the building of trusting relationships with staff in services is key, as is the importance of service user-directed goals and being accepted as a person [28, 33]. Participation of people who use drugs (peers) is an essential element of harm reduction services and one of its key principles [34, 35]. Services that are accessible, with staff who are good listeners and have caring, non-judgemental attitudes, can facilitate engagement with a range of population groups who can be reluctant to engage with mainstream services [36, 37] for a range of reasons. A very basic but essential point that has been highlighted in studies on harm reduction and substance use is that people should be treated like human beings with worth [27, 33] which is not necessarily what this group of people experience when they access health, specialist substance use or social services [38, 39]. The development of trusting, consistent and reliable relationships is also essential for those experiencing homelessness in order to facilitate access to services [27, 29, 33, 40, 41].

Neale and Stevenson interviewed people who were homeless with drug and alcohol problems living in hostels to examine the nature and extent of their social and recovery capital [42]. Participants viewed supportive relationships with professionals as critical to their wellbeing and future outcomes. Hostel staff were noted as going ‘above and beyond’ what was expected from them: being caring and responsive to needs and protecting people [42]. Developing good relationships between healthcare professionals and those who are homeless has also been found to be especially important for engagement, particularly when dealing with individuals with substance use and other health problems [22, 43]. Mills et al. interviewed staff working in homelessness primary care services in the UK and found that the development of trusting relationships, and listening to people well, was crucial to engagement of people experiencing homelessness [18]. Importantly, when people who are homeless developed good relationships with healthcare professionals, they would bring friends with them, thus extending reach [18]. Pauly has also highlighted the importance of trusting relationships as essential to access to primary care in Canada [44]. This literature has much in common with research on effective approaches for those experiencing homelessness and mental health problems highlighting the importance of flexible services, good relationships with professionals, care based on mutual communication and advocacy, practical support and having workers with lived experience [45]. Services viewed as unhelpful included those where staff were viewed as judgemental, lacking compassion and ‘clinically detached’, and used medical models of care. Refusing to give support because of substance use is also featured [45].

A recent development in homelessness settings is the use of a psychologically informed framework called psychologically informed environements (PIEs) to develop services for people with complex histories to enable *‘*the best chance of sustainably escaping the cycle of poor wellbeing and chronic homelessness’ [46]. The explicitly relational focus, working actively with a person’s experiences of trauma and ensuing emotional impact, lies at the core of PIEs [47], where the coping strategies that people develop to survive, including use of substances, are understood in this context. PIEs aim to help people to make changes to behaviours on their own terms using supportive relationships [47]. The five key areas of PIEs are developing a psychological framework, the physical environment and space, staff training and support, managing relationships and evaluation of outcomes [46]. Services implementing a PIE approach may change their reception areas to make them feel safer and more inviting, provide staff with opportunities for reflective practice and review eviction protocols [46]. Currently, there is limited research on PIEs, so this study will provide a novel and potentially unique empirical contribution to the field.

It is also important to acknowledge the vital contribution made by those with lived experience to relational interventions in the housing/homelessness and healthcare fields. Peer support workers have been successfully placed into mental health settings, and there is evidence that they can improve outcomes for those using services, particularly in terms of giving hope, facilitating empowerment and self-esteem [48, 49]. In terms of substance use, peers are involved in harm reduction and recovery services in a range of ways, including the provision of safer injection advice [10, 50]; running safe injecting sites, needle and syringe exchange and outreach programmes [51–54]; information about drug quality [55]; provision of take-home naloxone [10, 56]; and advocacy across a range of political and public arenas [57–59]. In their systematic review, Marshall and colleagues identified 36 different roles of peers in harm reduction initiatives, highlighting the diversity of involvement [60]. The involvement of peers in these services is considered to be highly beneficial in terms of facilitating engagement with services [31, 61]; increased access to, and engagement with, health and social care services and addiction treatment [52, 62]; adherence to antiretroviral therapy [63]; and reduced drug-related deaths [10] through the development of trusting relationships [61, 64–67]. Peer-delivered interventions have also been found to be effective, compared to more traditional outreach interventions, in reducing the risks associated with injecting drug use [68, 69]. Those who use drugs are willing and able to access peer-delivered services [70], and the peers offering services themselves report a range of benefits [71].

Peers have also been involved in research in the fields of substance use and homelessness [60]. Peer research has been argued to be ethically imperative, particularly in areas of social exclusion and potential objectification [72]. As Terry and Cardwell note: ‘Peer research is based on the assumption that shared experiences bring a unique quality to research; an understanding and empathy that results in a higher quality and more meaningful research process’ [73]. Accessible role models can help to challenge stigmatising views of people who use drugs and are homeless [74].

Peer support roles have also been developed and supported in homelessness settings in the UK (Cyrenians, St Mungo’s, Shelter and Groundswell), although rigorous evaluation is sparse [74]. O’Campo et al. examined the literature on community-based services for people who were homeless and experiencing mental health and substance use problems and found that, in one programme, peer support staff were particularly effective in developing good relationships with service users [43]. Research indicates that while peer workers benefit from their role in terms of increased confidence and self-esteem, and as a way of reintegrating into the community [64], there can also be challenges in terms of a lack of boundaries, power imbalances, stress, unclear roles, tensions over professionalism and dealing with challenging behaviours [48, 64, 75]. Effective training, supportive and reflective supervision and management, clear role descriptions and acceptable pay, are all important in addressing such challenges [48, 64, 75, 76].

Taken together, these studies highlight the importance of particular components of harm reduction that can contribute to engaging well with people who are marginalised within mainstream health, social and housing services. The critical component to both good engagement, and subsequent progress on self-identified life goals, seems to be the facilitation of trusting, supportive relationships where there are also practical elements of support provided, such as access to primary care and housing options. Non-judgemental attitudes are noted to be vital in engaging people with complex needs in healthcare, including those with problem alcohol and drug use experiencing or at risk for homeless. Despite a clear need for tailored harm reduction services for people who experience both homelessness and problem substance use, there is limited evidence regarding how harm reduction and health services are experienced by this group of people who are vulnerable to a range of health and social harms. The study described here aims to address this evidence gap.

### Study aims and objectives

**Fig. 1:**
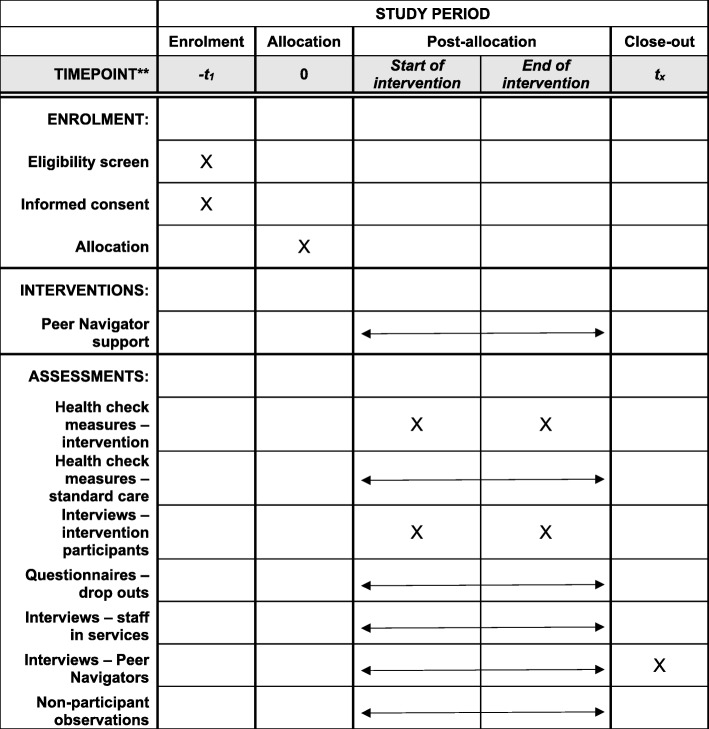
Standard Protocol Items: Recommendations for Interventional Trials

The SHARPS (Supporting Harm Reduction through Peer Support) study is a feasibility and acceptability trial of a relational peer-delivered intervention to support people who are homeless to address a range of health and social issues on their own terms. The study aims to examine whether it is feasible to deliver a peer-to-peer intervention (by ‘Peer Navigators’) based on PIEs that provides practical and emotional support for people experiencing homelessness and problem substance use in non-NHS settings. In this study, ‘Peer Navigators’ are those with lived experience of homelessness and/or problem substance use who are employed as specialist support workers to provide emotional and practical support to individuals and help them to engage with relevant services. Similar roles have been implemented elsewhere [77, 78]. Holistic health checks using standardised measures of physical and mental health, and measures of quality of life, relationships, and recovery, will also be conducted. It will assess the acceptability of the intervention, its ability to engage people, and appraise a range of critical intervention requirements that need to be fully understood before proceeding to a definitive trial. This article details the protocol of the SHARPS study and follows the recommendations of the Standard Protocol Items: Recommendations for Interventional Trials (SPIRIT) 2013 guidance [79] (please see Fig. 1 for the SPIRIT flowchart and Additional file 1: Table S1 for the SPIRIT checklist).

The study objectives are:

- To develop and implement a non-randomised, peer-delivered, relational intervention, drawing on principles of PIEs, that aims to reduce harms and improve health/wellbeing, quality of life and social functioning, for people who are homeless with problem substance use
- To conduct a concurrent process evaluation, in preparation for a potential randomised controlled trial (RCT), assess all procedures for their acceptability, and analyse important intervention requirements such as fidelity, rate of recruitment and retention of participants, appropriate sample size and potential follow-up rates, the ‘fit’ with chosen settings and target population, availability and quality of data and suitability of outcome measures.

The study will answer the following research questions:

- Is a peer-delivered, relational harm reduction approach accessible and acceptable to, and feasible for, people who are homeless with problem substance use in non-NHS settings?
- If so, what adaptations, if any, would be required to facilitate adoption in wider NHS and social care statutory services?
- What outcome measures are most relevant and suitable to assess the effect of this intervention in a full RCT?
- Are participants and staff/service settings involved in the intervention willing to be randomised?
- On the basis of study findings, is a full RCT merited to test the effectiveness of the intervention?

### Study design and methods

The intervention (health technology) being assessed in this feasibility and acceptability non-randomised study is a peer-delivered, relational intervention for people who are homeless, or at risk of becoming homeless, with problem substance use. The intervention, informed by the concept of PIEs, will provide practical and emotional support for a period of up to 12 months (between 2 and 12 months depending on the setting). Initially, support was to be provided for up to 12 months in all settings, but a small group of participants (*n* = 10) will receive a shortened intervention of 2/2.5 months due to one of the Peer Navigators leaving the role early. Peer Navigators will support participants to stabilise their lives through the development of trusting relationships alongside creating opportunities with them to access valuable resources that can also contribute to stabilisation.

The centrality of relationships within PIEs lies at the heart of our intervention. As there is limited research specifically on both PIEs and relationships, our use of this concept in the study will provide a novel and unique empirical contribution to the field. Peer Navigators will help people engage with services that are tailored to their health and social needs on their own terms, for example in ensuring that they are registered with GPs, dentists and optician services, if they wish to be, and receive appropriate assessments by such services. Importantly, the intervention will be co-produced in phase 1 of the study with a range of collaborators with selected expertise, and a detailed intervention guide created (more detail provided in the intervention section below).

When the relationship with the Peer Navigators has developed, and participants are willing, a holistic health check will be undertaken by researchers. The purpose of these measures (detailed below) is to capture an overall picture of the health of individual participants, ideally at two time points, and ascertain acceptability and completeness of the separate measures for a definitive trial. Onward referral to specialist services will be supported by the Peer Navigators, as required, and support will be offered for up to 12 months (between 2 and 12 months depending on the setting), and not withdrawn on the basis of continued problem substance use or abstinence. The comparator will be two standard care sites, and the health care check measures will be conducted by researchers with a sample of residents/service users in these settings. The study comprises three main phases.

Phase 1 (months 1–3) will address objectives 1 and 2:

1. Develop an intervention using co-production methods for use in community outreach/hostel settings
2. Create a manual to guide the intervention and an associated staff training manual

Phase 2 (months 4–21) is a non-randomised feasibility study that will deliver the co-produced intervention in six third sector intervention sites and address the following objectives:

3. Test the feasibility of recruiting to the intervention and measure the rate of recruitment/attrition in order to determine appropriate sample size and follow-up rates for a full RCT
4. Deliver a non-randomised, peer-delivered, relational intervention based on principles of PIEs, with integral holistic health checks (conducted by researchers) based on already identified outcome measures
5. Assess the acceptability and feasibility of all procedures in the intervention using normalisation process theory (NPT), including staff and participant perceptions of its value, strengths and challenges
6. Assess the acceptability of the holistic health checks/outcome measures, to determine the best way to measure outcomes for this particular intervention and population in a future RCT
7. Assess fidelity, adherence to the manual, ‘fit’ to context, and data availability and quality, and potential for wider adoption to NHS/statutory health and social care services.

Phase 3 (months 18–24) will involve the analysis and write-up of all study findings to address our research objectives 3, 5, 6, 7 and 8 (listed above), focusing on evaluating factors needed to deliver the intervention at scale. In addition, the intervention manual/training guidance will be refined to ensure that it is fit for purpose for a wider rollout in a range of settings, and for a definitive trial.

NPT [80] will provide a framework for the evaluation given that it is particularly suited to evaluating complex health interventions by providing a means of understanding and improving the way that interventions are implemented [80]. There are four NPT constructs: coherence, cognitive participation, collective action and reflexive monitoring [81]: Coherence refers to the process of understanding that individuals/organisations go through to either endorse or prevent an intervention being embedded into practice, cognitive participation involves enrolling and engaging individuals in the new practice, collective action is the work that individuals/organisations do to embed the new intervention into practice and reflexive monitoring refers to formal and informal appraisal of the new practice [80, 81]. In this study, NPT will facilitate analysis of how staff adopt/perceive the intervention, how those receiving it engage, how the Peer Navigators make sense of their role and other contextual factors impacting delivery. Using NPT will enable a better understanding of the intervention from the perspectives of all involved and inform a definitive trial.

### Settings

Three homeless outreach services in Lothian, Scotland, and three hostels in Liverpool and Bradford, England, were chosen for implementation of this intervention. All hosting services are non-profit, third sector housing organisations. To enable assessment of the differences between intervention and non-intervention care pathways, we have identified two standard care settings that are similar to the intervention sites, e.g. third sector/type of funding/types of staff roles and numbers in place/aims of service. Whether the settings are comparable will be explored in the process evaluation. As non-statutory, third sector services, developed to meet the needs of their specific populations, it will be highly unlikely that any service is completely comparable. In the two standard care sites, the same health check measures will be used with a sample of residents/service users in order to assess population differences and the acceptability of use of measures outside of the context of our intervention. We will also undertake non-participant observation in both intervention and standard care sites to document similarities/differences between care pathways. Interrogating the role of context will be key to our understanding of how the intervention works, most specifically in terms of the role of each of the services in hosting the Peer Navigators and the study and particular facilitators and barriers to the intervention.

### Recruitment and eligibility assessment

Recruitment to the intervention will be an ongoing process until the desired sample size of 60 participants is reached (see below for a discussion on sample size), or until the mid April 2019 (combining two trial recruitment strategies identified by Thoma et al. [82]). As described in the feasibility study conducted by Ferguson and Xie, for each participant that leaves the study, a new participant will be invited to engage in the intervention [83] until the ‘cutoff’ point of mid April 2019. In the event that there are more than 60 people who wish to take part, a waiting list will be kept so that if a participant drops out, a new person can be engaged until the ‘cutoff’ date.

To be eligible, participants must be currently homeless or at risk of homelessness, over the age of 18 years, self-report alcohol or drug problems (with participants themselves recognising that their substance use is a problem for them, prompted by questions such as ‘do you use alcohol or drugs? do you see this as a problem for you?’) and able to provide informed consent. The Peer Navigators will identify participants through the service in which they work, and from the outreach activities that they engage in, and from other referral points such as healthcare professionals and other agencies working in the local environment. A collaborative process will take place, to discuss the eligibility of each participant who is approached, between the Peer Navigator and the Service Managers in each of the study settings who line manage the Peer Navigator, in partnership with the study Chief Investigator and the study team.

Two Peer Navigators are based in three homelessness outreach services in Scotland and two are based in three hostels (Lifehouses) in the north of England. Two standard care sites have been selected, one in Scotland (third sector outreach service run by one of the providers of the intervention sites serving clients with similar needs) and one in England (third sector hostel run by one of the providers of the intervention sites serving clients with similar needs), that are comparable to the intervention sites in key ways (e.g. the needs of the client base, geographical area, staffing levels, organisational ethos and culture) but will not have a Peer Navigator located within them.

### Consent

All identified eligible intervention participants in the study intervention settings will be approached by the Peer Navigators and provided with a Participant Information Sheet and then asked to provide written informed consent at least 48 h later if they wish to join the intervention. Participants are advised that they might be approached at a later point regarding participating in the associated qualitative data collection (interviews) element of the study. This will be a separate process of providing information and then gaining informed consent; only a sub-set will be involved in this aspect of the study. These interviews will be conducted by Peer Researchers, who have been recruited and trained by the Scottish Drugs Forum (SDF). These individuals are separate from, and unknown to, the Peer Navigators. The Peer Researchers will have lived experience of problem substance use and will receive training in research methods, the study methods and the intervention. Interviews will also be conducted with staff in the intervention and standard care sites and with the Peer Navigators. (Examples of the Participant Information Sheets and consent forms are provided as Additional files 2 and 3).

Participants will be asked to state that they accept and understand limitations to confidentiality within the qualitative interviews and/or support discussions with Peer Navigators. These limitations refer to information about knowledge of harm or abuse to someone including the participant. If such disclosures do occur, this information will be shared with senior members of staff in each setting who will conduct a risk assessment as per their duty of care. In the event of this situation occurring, the person taking the disclosure will call the Chief Investigator, with an adverse event form being completed.

### Sampling, sample size and data collection

As this is a feasibility and acceptability study, no formal power calculation is required [84]. We aim to recruit 60 people to the intervention. This sample size was determined on the basis that, although sample sizes of between 30 and 50 have been recommended for feasibility studies [85, 86], we expect a degree of attrition because of the unstable nature of participants’ lives. In consultation with clinical colleagues, we slightly inflated our sample size based on the likelihood of 10–15% attrition to ensure participant volume [82]. While this sample is deemed a manageable caseload for the Peer Navigators, this will be carefully assessed as part of the process evaluation.

There are a number of different samples for data collection within the process evaluation:

- All consenting intervention participants (*n* = 60) will have quantitative data collected through the holistic health checks
- A purposive sub-sample (*n* = 20–25) of intervention participants will be asked to participate in semi-structured interviews (conducted by Peer Researchers), ideally at two time points if available/willing
- Participants who leave the study will be asked to complete a short questionnaire provided by Service Managers, which covers some key questions about their experiences in working with the Peer Navigator
- All four Peer Navigators (using criterion sampling) will participate in semi-structured interviews at three time points
- Service staff in both intervention and standard care settings (*n* = 16) will be identified using purposive sampling and asked to participate in semi-structured interviews

The purposive approach to sampling of the intervention participants for qualitative interviews will take account of the diversity of settings, gender, age, race/ethnicity, and the presence of disability or significant health concern, alcohol or drug use as main problem substance.

A small number of Peer Researchers will conduct qualitative semi-structured interviews with the sub-sample of intervention participants to examine their views on the intervention. Our team believes that participants in the intervention will be more comfortable speaking with someone who has been through similar life experiences and that this will lead to ‘richer’, more ‘valid’ data being gathered in relation to participant experiences of the intervention [87]. The Peer Researchers will be qualified partly on the basis of the lived experience of problem substance use and/or homelessness. They will be recruited by SDF from their wider pool of Peer Researchers, and although trained in research methods, they will be further trained in the SHARPS study methods and intervention. They will be actively supported by a User Involvement Officer from SDF on site during interviews. Interviews will take place where participants are willing/available at two time points, in the early to middle phase (January–April 2019) and then towards the end of the intervention (August–September 2019), to explore whether perceptions change over time.

Research team members will identify and recruit the wider service staff (*n* = 16) and participant (*n* = 20–25) samples at each of the six intervention sites and two standard care sites. The research team members will conduct all staff interviews. Staff samples will be purposive, based on diversity of setting, role within service, disciplinary background, gender and age. Qualitative interviews will be undertaken with all four Peer Navigators at three time points (beginning—June 2019, middle—April 2019 and end—November 2019) by research team members to assess changes in perception and practice. Please see Table 1 for more details on sampling, recruitment and data collection.

**Table 1:** Sampling, recruitment and data collection strategy

Sample	Recruitment strategy and timescale	Data collection methods and proposed sample size
1. Participants receiving the intervention	People who are homeless with problem substance use who are engaged with the intervention A sub-group of those in the intervention will be identified by researchers as the process evaluation sample for qualitative data collection. Timescale: months 9–11 and 16–17	As part of the holistic health check (*n* = 60), standardised measures of socio-demographic characteristics, housing status/quality and general health status; quality of life (SF36); substance use (SURE and MAP); mental health (GAD7 and PHQ9); will be used, plus measures to assess relationship quality (CARE). All participants will have this information collected if they consent to the health checks and the data collection component. The health checks will be conducted twice, towards the start and near the end of the intervention, by researchers. Individual face-to-face interviews (*n* = 20–25) conducted on two occasions by Peer Researchers approximately 20–40 min in duration. Interviews will examine various elements of feasibility and acceptability of intervention (except in Liverpool where only one interview will be attempted due to shortened intervention).
2. Participants who have dropped out	People who are homeless with problem substance use who initially engage but leave the intervention early, identified by the Service Manager and invited to complete a short, structured questionnaire. Timescale: as soon as possible after ending engagement.	All participants who drop out will be provided (via the Service Manager where the Peer Navigator is based) with a short questionnaire to voluntarily complete and return via a sealed envelope. This will ask questions about their experiences of working with the Peer Navigator and the reasons underlying their decision to withdraw their participation. This will not be shared with the Peer Navigator but go directly to the study Chief Investigator.
3. Peer Navigators	All four Peer Navigators employed for the duration of the project (training/intervention development, mid-intervention delivery and towards the end of the intervention period).	Individual interviews (*n* = 4) at three time points conducted by researchers (approx. 60–90 min). Interviews examine recruitment, health checks and potential outcome measures, training and support/supervision, fit to context, fidelity, acceptability—topics informed by feasibility study literature [73].
4. Service staff in intervention and standard care settings	Support workers, team leaders, managers/other staff working in the six intervention sites and two standard care settings. Timescale: months 6–12	Interviews (*n* = 16 across intervention/standard care sites), conducted by researchers (approx. 60 min) examining issues listed in box above, drawing on feasibility study literature on areas of focus noted above [73].
5. Intervention/standard care settings	Six intervention sites and two standard care sites will be included Timescale: months 6–12	Semi-structured, non-participant observations in all sites to gain an understanding of the of the culture and context of the settings, staffing levels, client group, activities provided, and fit between intervention and setting in the intervention sites.

### Intervention and intervention guide/manual

Another important dimension of this study is the co-production of the intervention at the start of the study. The intervention and intervention guide/manual will be co-produced by experts in homelessness, inclusion health, and PIEs and relational interventions; representatives from homelessness and third sector organisations; peers and people who have experienced homelessness and/or problem substance use via our Experts by Experience group and the Peer Navigators; and relevant health/medical professionals, following INVOLVE guidance [88]. We will do this by convening a full-day meeting to discuss the key components of the intervention and how these will be implemented. During this meeting, we will plan for the intervention end point in a way that is sensitive to the fact that participants who engage will have experienced many other relationship losses. We will aim to ensure that participants are connected to other support agencies before the intervention ends.

Following this full-day meeting, the study team will develop draft versions of the intervention and training manuals for circulation to all parties. We will work closely with all organisations and individuals, including the Peer Navigators, to ensure that these documents meet the needs of the target group, drawing on current literature and stakeholders’ own experience of working in the field and/or accessing services as a service user. The intervention manual will provide the Peer Navigators with the necessary information to carry out their roles, with detailed information about particular concepts and approaches; health and social issues affecting participants; study information; the intervention; and key contacts and local information. Service-based line managers of the Peer Navigators will also receive their own copies for reference. In line with other relationship-based therapies, manualisation of the intervention will be flexible rather than completely structured, as rigid approaches can reduce effectiveness, but will include instruction on PIEs, health check/measures and referral pathways. The manual/guidance will be further refined post-intervention and evaluation as one of the study outputs.

**Fig. 2:**
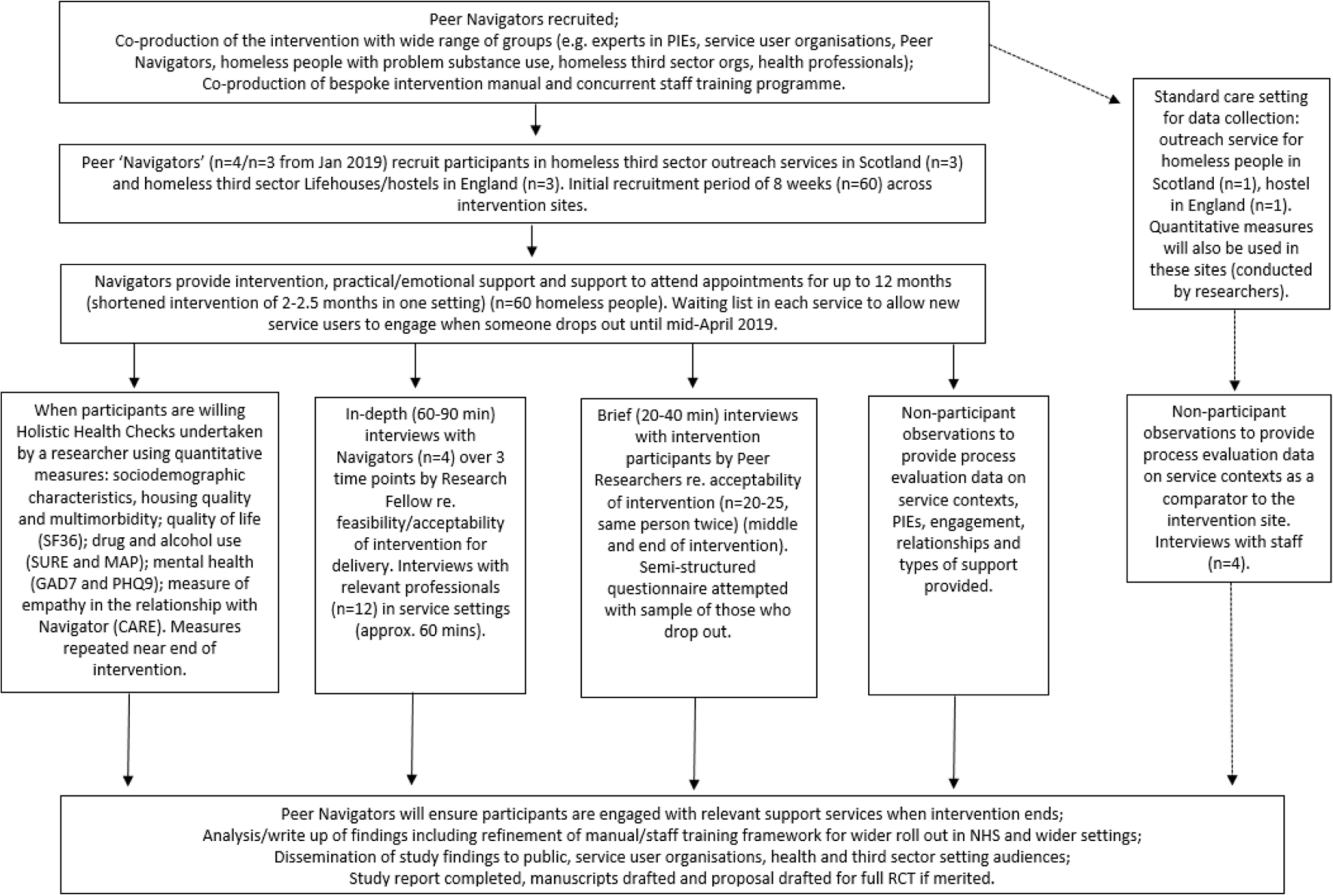
SHARPS study flowchart

Four part-time (30 h per week, 18-month contracts) Peer Navigators will be employed by the lead partner agency, paid on a Specialist Support Worker rate, and have the same terms and conditions as other staff in the organisation, including the right to continuing professional development as part of their roles. The Peer Navigators will provide a non-judgemental relationship and practical and emotional support for each recruited participant for a period of between 2 and 12 months, depending on the setting. The support provided will consist of support regarding the reduction of harms/potential for accessing treatment related to a person’s substance use, physical and mental health, housing, relationships with family, developing skills and hobbies, and training and education. The Peer Navigators will work with participants to identify these support needs and to put in place referrals, support to attend appointments, support to build relationships with new services and so on. As part of the proposal development work, the importance of practical support to attend appointments was highlighted by our experts by experience advisers consulted as part of the proposal development. In order to provide participants with this practical support, the Peer Navigators will have access to small amounts of petty cash held in the service settings. Support will be offered for up to 12 months (2–12 months depending on the setting), and not withdrawn on the basis of continued problem substance use or abstinence. Towards the end of the intervention, the Peer Navigators will have a conversation with participants to identify an ‘exit strategy’ in terms of their support needs in the months until and after the end of the intervention. Figure 2 shows the study flowchart.

**Fig. 3:**
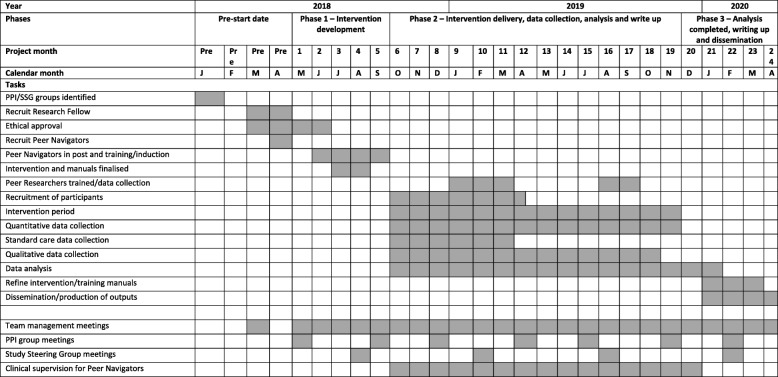
SHARPS study detailed timeline

### Training in the intervention and fidelity

The Peer Navigators will receive induction and advanced ‘front loaded’ training in the first 3 months in their post and will receive updates as appropriate throughout the study. Training will be in areas related to the intervention such as harm reduction, the relevance of a trauma to substance use behaviour, professional boundaries and therapeutic relationships, and PIEs. They will also receive training and induction to the study, including on recruitment and relevant ethical issues such as assessing eligibility and informed consent. A training manual will be produced and refined as part of the study. Fidelity to the guide and core components of the intervention will be assessed in the interviews with Peer Navigators. It is important to formally acknowledge that the study team understands that each Peer Navigator will bring their own experiences and individuality to the intervention and to each of their relationships with participants and the feasibility design allows a diversity of approach to be well explored and reflected upon. Each Peer Navigator will receive regular one-to-one (face-to-face or phone) clinical supervision with a Clinical Psychologist with expertise in working with the participant group and supporting staff working with this group. The value of this supervision will be explored in the process evaluation.

### Outcome measures via ‘holistic health checks’

The following outcome measures will be administered as two ‘holistic health checks’, one in the first few months of the intervention (November 2018–May 2019) and one near the end (August–October 2019).

- Socio-demographic characteristics, housing status/quality and general health status will be assessed and cover demographics, education, housing, health conditions, medication, health status and future service use.
- Mental health will be measured using the Generalised Anxiety Disorder 7 (GAD7), a 7-item tool covering symptoms of anxiety [89], and the Patient Health Questionnaire 9 (PHQ9), a 9-item tool covering symptoms of depression [90].
- Substance use will be measured using the Maudsley Addiction Profile (MAP), which has been slightly amended to fit the current population (it will not be scored). The 36-item tool covers substance use (type/frequency/method), overdose, treatment, injecting and sexual behaviour, physical and psychological health, social functioning, relationships and illegal activities [91]; and the Substance Use Recovery Evaluator (SURE), a 26-item tool covering drinking and drug use, self-care, relationships, material resources, outlook on life and importance of previous items [92].
- Health-related quality of life will be measured using the Short-Form 36 (SF36), a 36-item tool covering physical and emotional health status, the effect of health on daily activities and social activities and experiences of pain [93].
- The quality of the relationship between the Peer Navigator and the participant will be measured using the CARE Patient Feedback Measure, a 10-item tool assessing empathy in the context of relationship [94].

These holistic health checks will take place in a private space with the researchers taking sufficient time to work through each of the measures with each participant allowing time for full discussion if needed. The measures will be paper-based and conducted in a sensitive and relational manner. The Peer Navigators will be available throughout if the participant wishes to have their support.

### Data analysis

Raw data and overall scores from each of the outcome measures will be inputted from the paper-based measures into Statistical Package for the Social Science (SPSS) and analysed using descriptive statistics to gain an understanding of population characteristics in both the intervention and standard care settings, as well as to assess process issues in the collection of these data such as missing/incomplete and poor quality data. Analysis using descriptive statistics is appropriate in feasibility studies [95].

The framework method [96] will be used for the management and analysis of all qualitative data (interview transcripts and observational fieldnotes) because of its ability to support the analysis of the eight different settings (six intervention, two standard care) as cases and because it allows within case and between case comparisons [96]. Framework involves five stages as follows: (1) familiarisation, where the transcripts are read multiple times; (2) identifying a thematic framework, whereby the researchers recognise emerging themes in the dataset; (3) indexing, which involves identifying data that correspond to a theme; (4) charting, in which the specific pieces of data are arranged in tables according to themes; and (5) mapping and interpretation, involving analysis of key characteristics in the tables and providing an interpretation of the dataset [96]. The observational data will be gathered in the form of semi-structured fieldnotes using a pro-forma.

All qualitative data will be analysed with the support of the computer software package NVivo. The staff interviews from the settings will be analysed together and will compare intervention/non-intervention differences. The interviews from Peer Navigators will be analysed together with particular issues specifically noted, e.g. fidelity to the intervention, challenges with recruitment and drop out, fit between intervention and contexts. We will collect data at different time points with both Peer Navigators and participants, and data analysis will specifically interrogate whether, and how, perceptions of the intervention, its challenges and benefits, changed over time. Data analysis will be iterative throughout phases 1 and 2, supported by the use of NPT [80] to identify contextual influences on the implementation of the intervention across the different settings.

A sub-group of Peer Researchers will be invited to participate in the data analysis and interpretation, supported by the study team. They will be provided with an anonymised selection of interview transcripts and asked to provide their interpretations of the themes arising and their significance (stages 1–3). We will also involve the Peer Researchers in stage 5 of the analysis, as a form of ‘member checking’ to enhance the validity and trustworthiness of the study findings [97].

The quantitative data from the outcome measures will be combined with the qualitative data from the staff, Peer Navigator and participant interviews and observational fieldnotes, using concurrent triangulation design [98]. This design has a single-phase timing and generally involves the concurrent, but separate, collection and analysis of quantitative and qualitative data. Data sets are merged, typically by bringing separate results together in the interpretation or by transforming data to facilitate integrating the two data types during analysis. The analysis will address all the research questions including whether and how a future RCT should be conducted to test effectiveness.

### Trial registration

The trial is registered with the ISRCTN registry. The trial reference is ISRCTN15900054.

## Discussion

This study will provide evidence regarding the feasibility and acceptability of a peer-delivered harm reduction intervention to people experiencing homelessness and problem substance use. While there is considerable evidence regarding harm reduction approaches more generally, the evidence is lacking for approaches that are both acceptable to, and effective for, people who are homeless who use both drugs and alcohol. There is also limited evidence regarding peer support interventions with this population. As above, relational and peer-led harm reduction approaches show much promise in engaging, and then effectively working with, people with intersecting housing, health and alcohol and drug use challenges to reduce harms and enhance physical and mental health and wellbeing, quality of life, social functioning and relationships. While there is evidence that both the relational ‘therapeutic alliance’ and the peer-led approach can be powerful tools for this vulnerable population, more rigorous investigation is required as evidence to date is based on small scale pilot studies.

This feasibility and acceptability study aims to address this gap and inform a decision on whether or not to proceed to a definitive trial. Success will be measured in terms of recruitment and retention of participants, the suitability of outcome measures and positive experiences of participants, Peer Navigators and staff. A key consideration in the design of a future RCT is the settings in which the intervention is delivered. This feasibility and acceptability study is being conducted in third sector settings, covering both residential and outreach environments. In planning a future definitive RCT, decisions will need to be made regarding whether any adaptations are required to facilitate adoption in wider NHS, social care statutory services and statutory homelessness services. Furthermore, it would be necessary to determine whether randomisation would be feasible and acceptable for participants, staff and services in a future trial.

## Additional files

Additional file 1: SPIRIT 2013 checklist: Standard reporting items for intervention trials. (DOC 139 kb)

Additional file 2: Supporting Harm Reduction through Peer Support (SHARPS). Consent form (Intervention Participants). (DOCX 44 kb)

Additional file 3: Supporting Harm Reduction through Peer Support (SHARPS). Participant Information Sheet (Intervention Participants). (DOCX 111 kb)

## Abbreviations

GP: General Practitioner
NPT: Normalisation process theory
PIEs: Psychologically informed environments
RCT: Randomised controlled trial
SPIRIT: Standard Protocol Items: Recommendations for Interventional Trials
SPSS: Statistical Package for the Social Science
