# Supplementary material for: Supporting Harm Reduction through Peer Support (SHARPS): testing the feasibility and acceptability of a peer-delivered, relational intervention for people with problem substance use who are homeless, to improve health outcomes, quality of life and social functioning and reduce harms: study protocol
Source: Pilot Feasibility Stud. 2019 Apr 29;5:64. doi: 10.1186/s40814-019-0447-0 (PMC6489271; doi:10.1186/s40814-019-0447-0)
Supplement: Supplementary file 2 — Supporting Harm Reduction through Peer Support (SHARPS). Consent form (Intervention Participants). (DOCX 44 kb) [file 40814_2019_447_MOESM2_ESM.docx]

**
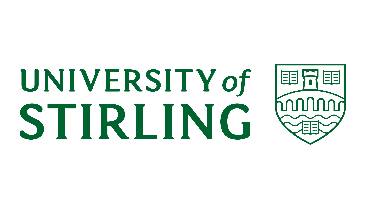
**

**Supporting Harm Reduction through Peer Support (SHARPS)**

**Consent form (Intervention Participants)**

Please read each of the statements below. If you have any questions please ask the Peer Navigator or someone from the study team. Please tick to confirm that you’ve done this and only sign the form when you are happy with ALL statements.

All data collected during the intervention will be stored in a safe and secure location. The Peer Navigator will collect information needed to provide you with support but it might need to be shared with the Service Manager and other staff in the service, or the other Peer Navigator when covering absences. Only members of the study research team will have access to the anonymised whole person health check data.

By signing this form, you agree to take part in a ‘peer-delivered’ intervention providing support and a whole person health check. You will be given a copy of this form to keep.

| 1. I confirm that I have read and understand the participant information sheet for the above study. I have had the opportunity to consider the information, ask questions, and have had these answered properly. |  |
| --- | --- |
| 1. I understand that my participation is voluntary and I am free to withdraw at any time, without giving a reason, and without my care rights being affected. |  |
| 1. I understand that the information I provide will be accessed by my Peer Navigator and the Service Manager for support reasons and might be shared with another member of staff if they are covering absences. |  |
| 1. I understand that any personal data collected about me will be collected for research purposes, will be kept for only as long as it is necessary, and handled in accordance with data protection legislation. |  |
| 1. I agree that while all information will be kept confidential, the Peer Navigator or study researchers will break confidentiality if they feel that either I, or another person, is at immediate risk of being harmed or abused. |  |
| 1. I understand that the anonymised research data will be published at the end of the study. |  |
| 1. I agree to my anonymised health check data being collected and stored by members of the study team at the University of Stirling. |  |
| 1. At a time that I feel comfortable to do so, I agree to participate in a health check towards the beginning of the study. |  |
| 1. At a time that I feel comfortable to do so, I agree to participate in a health check towards the end of the study. |  |
| 1. I agree to participate in the SHARPS study. |  |

Name of Participant Date Signature


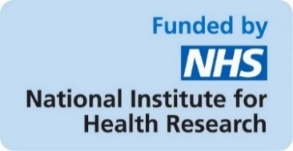


Name of Peer Navigator Date Signature
