# Supplementary material for: Supporting Harm Reduction through Peer Support (SHARPS): testing the feasibility and acceptability of a peer-delivered, relational intervention for people with problem substance use who are homeless, to improve health outcomes, quality of life and social functioning and reduce harms: study protocol
Source: Pilot Feasibility Stud. 2019 Apr 29;5:64. doi: 10.1186/s40814-019-0447-0 (PMC6489271; doi:10.1186/s40814-019-0447-0)

**
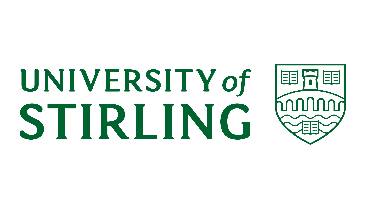
**

**Supporting Harm Reduction through Peer Support (SHARPS)**

**Participant Information Sheet (Intervention participants)**

**Key information**

We are researchers based at the University of Stirling conducting a study on harm reduction through peer support. The study involves a member of staff supporting participants to address their social and health problems in a supportive way. This member of staff has also had personal experience of homelessness and/or using alcohol or drugs in a way that has had a negative effect on their lives.

**Who can take part?**

To take part you need to be:

- 18 years old or over;
- Currently using drugs and/or alcohol in a way that you feel has a negative effect on your life;
- Currently homeless or at risk of being homeless.

Before you decide whether you would like to take part of not, we would like to explain why this study is being conducted, and what your involvement would entail. We have provided the names and details of different people involved in the study throughout this sheet –please speak to them if you have any questions and they will be happy to answer them.

**What is this study about?**

Here is some background information:

- People who are homeless often experience physical and mental health problems. This often includes using drugs or alcohol in a way that has a negative effect on their lives.
- Harm reduction aims to support people ‘where they are at’, rather than trying to get people to change before they are ready or able to do so. It aims to improve people’s quality of life overall.
- There’s a lack of research on the best way of doing this. However, of the research that exists, trusting relationships with staff have been identified as being very important in helping people with these problems.
- In this study, we are aiming to explore how this approach works in practice and to see if individuals with these problems value are getting this type of support.

**Who is organising and funding the study?**

The SHARPS study has been set up by a team from the University of Stirling, the Scottish Drugs Forum, NHS Lothian and the University of Aberdeen, in collaboration with The Salvation Army, Streetwork and the Cyrenians who run the services where the study will be based.

Your involvement in the study will be for one year but the study is funded for two years in total. Funding has been provided by the National Institute for Health Research (NIHR).

**Who has approved the study?**

All research at the University of Stirling is reviewed by an independent group of people called a Research Ethics Committee which is there to protect your safety, rights, wellbeing and dignity. The study has been reviewed and was given a favourable review by the University of Stirling’s NHS, Invasive or Clinical Research Committee (NICR), and The Salvation Army’s Research Ethics Group.

**What does taking part involve?**

- Our study will involve **Peer Navigators** who will provide practical and emotional support to people who are homeless and have drug and/or alcohol problems.
- **‘Peers’** are people with personal experience of these issues. They are recovering and have been trained to provide support in a range of areas.
- The word **‘Navigator’** means that these workers will closely support each person. The Peer Navigators will work with you to try to help you improve your quality of life and health in a safe and supportive environment.
- As part of the study, a **‘whole person’ health check** (via questionnaires) will be offered at the beginning and towards the end. Checks like this can take a while and can be off-putting so we will make sure that it is offered when you are comfortable with it. This health check will be conducted with a researcher, and the Peer Navigator, if you would like them to be there. The researcher will ask you questions about your physical and mental health.
- The Peer Navigators will help you to find **services that can meet your needs**, such as healthcare, housing or counselling. They will also go with you to appointments if needed. **Practical support** will also be available, ensuring you have enough money to travel to appointments and to buy basics such as hot drinks, food and bus fares.

**Who are the Peer Navigators?**

There are two Peer Navigators based in this service who will provide this support [Peer Navigator 1 and Peer Navigator 2]. Both have been trained to carry out this role and have experience of working in this area already, including supporting people to access services.

| **[Peer Navigator 1] (Lead in service)**  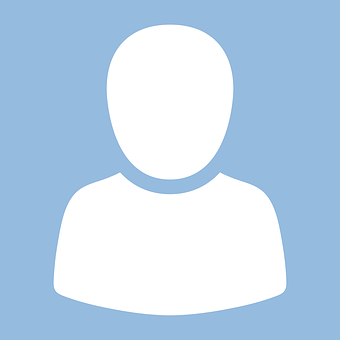 | **[Peer Navigator 2]**  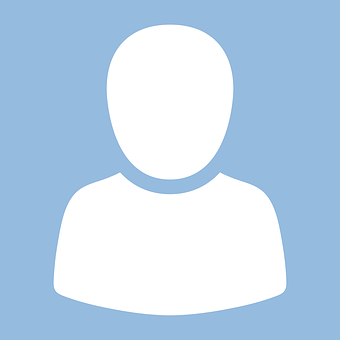 |
| --- | --- |

If you take part in this study, you will work with one of these Peer Navigators most of the time. However, there may be times (e.g. when your Peer Navigator is away) when you will work with the other Peer Navigator based in this service.

**The intervention will last for a maximum of 12 months**

**What happens to me after 12 months?**

This is a research study only funded for a set period of time. The support you will receive from the Peer Navigator will come to an end after 12 months. While you are working with the Peer Navigator they will support you to develop relationships with other services. We hope that this support will mean that services are able to better meet your needs so that when the intervention period ends you are in a much stronger position. The Peer Navigator will aim to ensure that others are there to provide support on an ongoing basis (not time limited) and that everyone is prepared for the ending.

**What else might I be asked to do?**

You might be asked by the Peer Navigator if you would like to participate in one or two short and informal discussions (interviews) about your experiences of the intervention. This is because we have to evaluate the intervention and talk to different people involved to do this. One of these will take place during the early part or middle of the intervention, and one towards the end.

You do not have to participate in these discussions if you do not want to. You can participate in one or both of these, the choice is yours.

All of these discussions will be conducted by ‘Peer Researchers’ who also have personal experience of homelessness and/or problem drug/alcohol use. They have been trained to carry out discussions in studies like this one. Information from these discussions will not be passed onto the Peer Navigator.

If you have any questions about these discussions, please speak to the Peer Navigator or the Service Manager. The Peer Navigator might talk to you again about these, when you’re both settled into the intervention.

You will be asked to separately if you wish to take part in these discussions. There is a separate consent form too.

**Do I have to take part in the intervention?**

No, it is completely up to you. If you decide to take part in the intervention you will be asked to sign a consent form. You are free to stop taking part in the intervention at any time, without giving a reason. Your decision has no influence on the care and support you currently receive from other service providers.

**Are there any benefits for me in taking part?**

We hope that people taking part in the study will benefit from the support received from the Peer Navigator.

Working with the Peer Navigator for up to 12 months may help you to improve:

- Your physical and mental health;
- Your housing situation;
- Access to services (such as GP, dentist, optician);
- Your awareness of local services that might be able to help you;
- Your awareness of particular health conditions and how to manage them.

We cannot guarantee that you will experience any or all of these, but we hope that working with the Peer Navigator will be helpful to you.

**Are there any risks for me in taking part?**

We hope that your participation in the intervention will not cause you any harm and we have tried to minimise any harmful effects of taking part, such as the intervention coming to an end after one year. During the ‘whole person’ health checks you will only have to answer questions and no tests (such as bloods) will be carried out. All the questions are used to help work with you to provide the support you need.

Answering personal questions may evoke feelings in you - you will be reminded that you only have to answer the questions you want to, and at a time you feel comfortable. If you find any question upsetting, the researcher and the Peer Navigator will be there to support you and, if needed, help you get some additional support from somewhere else.

**What information will you collect about me as part of involvement in the intervention?**

The Peer Navigator will collect some basic information about you, including your name and date of birth, only if you want to give this information. During the health checks, the researcher will collect some information about your health. All data will be kept confidential and only accessed by the Peer Navigator (including the other Peer Navigator who may provide cover from time to time), the Service Manager (and his/her deputy if absent) and some members of the research team. You will be asked about things like:

- Personal details and circumstances (including gender, marital status, ethnic origin, education level and housing situation);
- Quality of life, including health in general and relationships with others;
- Anxiety and depression;
- Alcohol and drug use.

The Peer Navigator might also collect information that is relevant to your particular needs, but they will always ask for your permission to do so.

**Will the information be kept confidential and be anonymised?**

All data will be stored in a secure location and will be kept confidential. Only the Peer Navigators and a small group of individuals from the study team will have access to it. This personal information will be kept for only as long as is necessary. When we write up the findings from the study, it will not be possible to identify you.

Due to the nature of this work, the Peer Navigator will share some details about you with the Service Manager or his/her deputy to ensure that they are providing you with the right types of support. If there are particular things you do not wish to be shared, please tell the Peer Navigator.

The Peer Navigators will not tell anyone else (other than the Service Manager or his/her deputy) about what you’ve told them unless:

1. You have given them permission to; or
2. You have told them something that concerns yourself or another person being at immediate risk of harm or abuse. In these situations, the Peer Navigator has a legal duty to break confidentiality and inform staff in the service and the study lead.

We will ensure we abide by data protection legislation. We have provided more information about how we will keep your information safe at the end of this document under ‘Data Protection Privacy Notice’.

**What will you do with the findings from the study?**

The basic information you provide to us (such as gender, ethnic origin and housing situation), and information from the health checks, will be included in the evaluation of the overall intervention. These findings will be published in a research report to the research funder. The findings will also be published in academic journals. We will provide you with a copy of the study findings if we are able to stay in touch after the study ends – please let us know the best way of doing this. Selected findings will be also presented at conferences and in on-going blog posts. We won’t use any personal details in any of the study reports.

**What should I do if I want to take part?**

If you would like to join the intervention, then all you need to do is to tell the Service Manager or speak to the Peer Navigator.

You will have the opportunity to ask any further questions that you might have.

**Who should I go to if I’m concerned about the study in some way?**

If you have a concern about any aspect of this study, you should speak to the Peer Navigator who will do their best to answer your questions. Alternatively, please contact the Study Lead (please see all contact details below). If you are still unhappy and want to speak to someone independent of the study, you can do this by contacting Professor Alison Bowes (Dean of Faculty of Social Sciences, University of Stirling [a.m.bowes@stir.ac.uk](mailto:a.m.bowes@stir.ac.uk)). The service staff can help you to make contact if needed.

**What happens if I take part in the study but later change my mind?**

If you take part in the intervention, but later decide you no longer want to be involved, you need to let one of the Peer Navigators know, or a service manager or member of the study team. If you decide not to be involved any more, it will not be possible for you to re-join the study. At this stage, we would ask you to complete a short questionnaire to learn more about your experiences of working with the Peer Navigator. Your decision to leave the study would be fully respected and you are not required to complete the questionnaire if you don’t want to.

**Service contact details**

**Peer Navigator (Lead in Service) contact details**

**Peer Navigator contact details**

**Study lead, SHARPS** **study contact details**

**Data Protection Privacy Notice**

*The data controller for this project will be the University of Stirling. The University of Stirling's Data Protection Office provides oversight of the University's activities involving the processing of personal data. The University’s Data Protection officer is Joanna Morrow, Deputy Secretary. If you have any questions relating to data protection these can be addressed to:* [*data.protection@stir.ac.uk*](mailto:data.protection@stir.ac.uk) *in the first instance. The legal basis for the processing of your personal information is that it is necessary for the performance of a task that is in the public interest. Your personal data will be processed only so long as is required for this research project. If we are able to anonymise or pseudonymise the personal data you provide we will undertake this, and will endeavour to minimise the processing of personal data wherever possible.*

**Thanks for taking the time to read this and please feel free to take this away with you.**


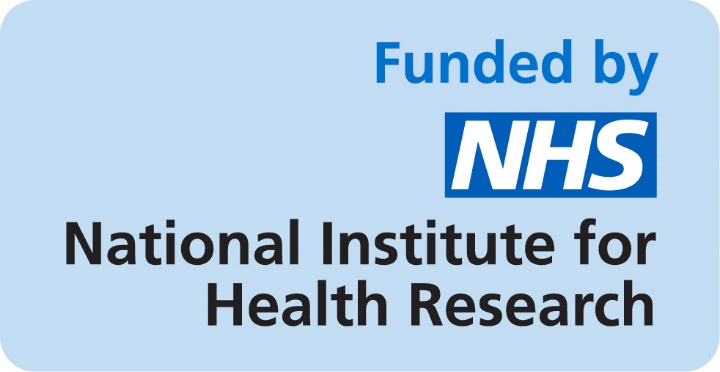

Supplement: Supplementary file 3 — Supporting Harm Reduction through Peer Support (SHARPS). Participant Information Sheet (Intervention Participants). (DOCX 111 kb) [file 40814_2019_447_MOESM3_ESM.docx]
